# Study on fresh processing key technology and quality influence of Cut Ophiopogonis Radix based on multi-index evaluation

**DOI:** 10.1515/biol-2022-0638

**Published:** 2023-07-19

**Authors:** Xiaoyang Cai, Hongmei Deng, Wenjing Li, Hongyan Li, Min Li

**Affiliations:** Key Laboratory of Standardization of Chinese Herbal Medicine, State Key Laboratory Breeding Base of Systematic Research, Development and Utilization of Chinese Medicine Resources, Ministry of Education, Chengdu University of Traditional Chinese Medicine, Chengdu 611137, China

**Keywords:** Ophiopogonis Radix, fresh processing, cut system, multi-indices, processing technology

## Abstract

The purpose of this study was to ascertain the fresh processing technology of Cut Ophiopogonis Radix using a multi-index evaluation. This study comprehensively evaluated the fresh processing technology of sliced Cut Ophiopogonis Radix by investigating the cutting methods, cutting thickness, and drying conditions, and referring to The Chinese Pharmacopoeia 2020 edition. The appearance traits, internal quality (extract, total saponins, total flavonoids, total polysaccharides), and drying efficiency were used as evaluation indexes. The physical attributes of Cut Ophiopogonis Radix were found to vary based on the processing techniques employed. The shape, surface characteristics, texture, and color were observed to differ across the different methods. Notably, the apparent quality of Cut Ophiopogonis Radix was superior in samples processed using A_1_B_1_C_1_, A_1_B_2_C_2_, and A_3_B_1_C_3_ techniques. Drying time and energy consumption of Cut Ophiopogonis Radix produced by the A_1_B_2_C_2_ and A_2_B_1_C_2_ processes were less than those of other treatments, making them the optimal process for fresh processing Cut Ophiopogonis Radix. The impact of the cutting method and thickness on the extract was found to be statistically insignificant (*P* > 0.05). However, the drying method was observed to have a significant impact on the extract (*P* < 0.05). The cutting method, Cut thickness, and drying method did not affect the total saponin content (*P* > 0.05), but they had significant effects on the total polysaccharide and flavonoid contents (*P* < 0.01). Total polysaccharides were most affected by the cutting method, while total flavonoids were most affected by the drying condition. Based on the characteristics and internal quality, the fresh processing technology for Cut Ophiopogonis Radix was determined: fresh Ophiopogonis Radix was sliced to a thickness of 2–4 mm and dried at 55°C or a low temperature. The feasibility of Cut Ophiopogonis Radix is improved through its fresh processing. According to the evaluation indices, it is recommended to utilize the novel processing technique involving “fresh Ophiopogonis Radix” with fresh cuts, a cut thickness ranging from 2 to 4 mm, and drying at a temperature of 55℃ or through low-temperature drying. The Cut Ophiopogonis Radix exhibited favorable appearance and internal characteristics, thereby furnishing a scientific basis and innovative insights for the production of ophiopogon decoction slices.

## Graphical abstract

**Figure:**
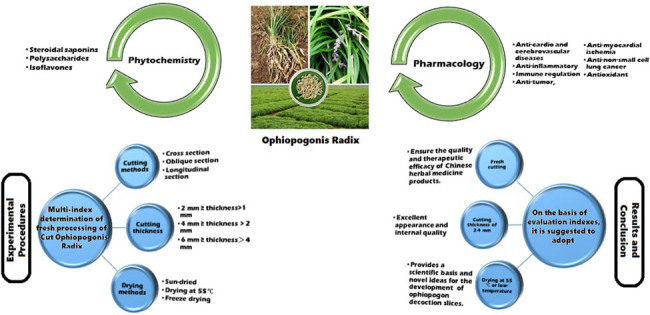

## 1 Introduction

Ophiopogonis Radix is the dried tuberous root of *Ophiopogon japonicus* (L.f.) Ker-Gawl. of the Liliaceae plant, which has the properties of nourishing yin and promoting fluid, moistening the lungs, and clearing the heart [1]. Ophiopogonis Radix is frequently utilized as traditional Chinese medicine. The earliest documentation of this herbal medicine can be traced back to “Shennong’s Classic of Materia Medica,” which is regarded as the highest standard and includes records of herbal medicines from various dynasties. Ophiopogonis Radix is a well-known, authentic medicinal substance that is mostly produced in the provinces of Sichuan and Zhejiang in China. It is composed of Zhejiang Ophiopogonis Radix and Sichuan Ophiopogonis Radix, respectively. Based on statistical data, it can be inferred that Ophiopogonis Radix serves as the primary ingredient in 573 Chinese patent medicine prescriptions and 959 other prescriptions. During the processing of medicinal materials into decoction pieces, various issues may arise, including challenges in the cutting process, overlapping and duplication of production procedures, loss of active ingredients, complications in separating non-medicinal components, difficulties in drying and storing, escalated processing expenses, and vulnerability to secondary pollution [2,3]. At the same time, in conjunction with the features of each medicinal material, the 2015 edition of “Pharmacopoeia of The People’s Republic of China” specifies 64 medicinal materials that can be freshly processed at the site of origin. The 2020 edition of the “Pharmacopoeia of The People’s Republic of China” [1] has expanded its coverage to encompass 69 species, except for Ophiopogonis Radix. Furthermore, it has been demonstrated that fresh processing is feasible and applicable for various medicinal materials, including Chuanxiong Rhizoma [4], Asparagi Radix [5], Gastrodiae Rhizoma [6], Salviae Miltiorrhizae Radix et Rhizoma [7], Notoginseng Radix et Rhizoma [8], and Cinnamomi Cortex [9], among others. The “Plan for the Protection and Development of Chinese Medicinal Materials (2015–2020)” clearly recommends the implementation of “fresh processing and intensive processing” of medicinal materials. The provinces of Yunnan, Shandong, and Gansu simultaneously introduced fresh processing varieties. For instance, Angelicae Sinensis Radix, Codonopsis Radix, Astragali Radix, Hedysari Radix, Glycyrrhizae Radix et Rhizoma, Rhei Radix et Rhizoma, and Isatidis Radix are among the first batch of fresh processing varieties released by Gansu Province. Documents were issued by Hubei Province and Hebei Province to authorize the procurement of freshly processed Chinese herbal medicines by manufacturers of Chinese herbal medicine. The emergence of fresh processing can be attributed to the modernization and industrialization of traditional Chinese medicine.

The processing of traditional Chinese medicine is a significant aspect of clinical medicine within the realm of traditional Chinese medicine. China has a rich historical tradition of utilizing traditional Chinese medicine, with documented instances of fresh processing and utilizing freshly picked medicinal substances. For example, in the Han Dynasty [10,11,12] (Zingiberis Rhizoma Recens, Anemarrhenae Rhizoma, and Paeoniae Radix Alba), Jin Dynasty [13] (Mori Cortex and Imperatae Rhizoma), Nanqi [14] (Angelicae Dahuricae Radix, Peucedani Radix, etc.), Liang Dynasty [15] (Zingiberis Rhizoma Recens, Ophiopogonis Radix, and Belamcandae Rhizoma), Tang Dynasty [16,17,18,19] (Rhei Radix et Rhizoma, Eucommiae Cortex, Phytolaccae Radix, etc.), Song Dynasty [20,21,22,23] (Rosae Laevigatae Fructus, Angelicae Sinensis Radix, etc.), Yuan Dynasty [24,25] (Zingiberis Rhizoma Recens, Quisqualis Fructus, Mume Fructus, etc.), Ming Dynasty [26,27] (Anemarrhenae Rhizoma, Atractylodis Rhizoma, Angelicae Sinensis Radix, etc.), Qing Dynasty [28,29,30] (Zingiberis Rhizoma Recens, Atractylodis Rhizoma, etc.) all have records of fresh processing of the origin of some medicinal materials. The fresh processing of certain varieties, such as Angelicae Sinensis Radix, Rhei Radix et Rhizoma, Phytolaccae Radix, Eucommiae Cortex, etc., is predominately based on the fresh cut system, which is still in use today. Previous editions of the “Pharmacopoeia of the People’s Republic of China” describe “removing impurities, washing, moisturizing, rolling, and drying” as the steps involved in the processing of Ophiopogonis Radix pieces. The preparation process of Ophiopogonis Radix necessitates additional processing of the medicinal materials, resulting in increased labor and time requirements, ultimately leading to higher production costs. On the other hand, the therapeutic elements in the medicinal materials will be lost during the infiltration process. It is vital to find a practical solution for the issue of scientifically processing Ophiopogonis Radix fragments. Indicators such as total polysaccharides and total flavonoids were introduced to the quality control indicators of the 2020 edition of the “Pharmacopoeia of the People’s Republic of China” as a result of this study. Multi-indices were utilized to assess the fresh processing technology and conventional technology of Ophiopogonis Radix origin. The present investigation aimed to examine the scientific validity, practicality, and relevance of the method used for the fresh processing of Ophiopogonis Radix. The primary objective was to reduce the processing time, conserve energy, and preserve the high quality of Ophiopogonis Radix.

## 2 Materials

### 2.1 Instruments

A580 UV-Vis Spectrophotometer (Aoyi Instruments (Shanghai) Co., Ltd); Milli-Q Advantage A10 ultrapure water meter (Merck Millipore, France); BP121S 1/100,000 electronic balance (Sartorius, Germany); KQ-500VDE Dual Frequency CNC Ultrasonic Cleaner (Kunshan Ultrasonic Instrument Co., Ltd); DUG-9070B intelligent electric heating constant temperature blast drying oven (Shanghai Langgan Experimental Equipment Co., Ltd); CHRIST ALPHA 1-4 LSC freeze-dryer (Matian hrist freeze-dryer GmbH, Germany).

### 2.2 Reagents

The fructose reference material was obtained from Sichuan Vikki Biotechnology Co., Ltd (batch number wkq16062201, mass fraction ≥98%). Sichuan Vikki Biotechnology Co., Ltd supplied the hesperidin reference material (batch number wkq15123105, mass fraction ≥98%). Ruscosapogenin reference substance (batch number OVTX-UC34, mass fraction ≥98%) was obtained from the China Institute of Food and Drug Inspection. Chengdu Kelong Chemical Reagent Factory provided 95% ethanol, methanol, *n*-butanol, and ammonia test solutions of analytical grade. Perchloric acid was from Tianjin Zhengcheng Chemical Co., Ltd; Anthrone from Shanghai Kefeng Chemical Reagent Co., Ltd; concentrated sulfuric acid of analytical grade from Sichuan Xilong Chemical Co., Ltd; and distilled water was from Youpu series ultrapure water device.

Fresh Ophiopogonis Radix products were purchased from the Ophiopogonis Radix planting base in Luxi Town, Santai County, Sichuan Province. Professor Li Min of the Chinese Medicine Appraisal Department at Chengdu University of Traditional Chinese Medicine determined that it was the fresh tuberous root of *Ophiopogon japonicus* (L.f.) Ker-Gawl., a genus of the Liliaceae family.

## 3 Methods

### 3.1 Processing methods

After removing fibrous roots and contaminants, cleaning, and drying the surface moisture, fresh Ophiopogonis Radix was cut by hand. Using the method of cutting (A), the thickness (B), and the method of drying (C) of fresh slices as factors, three levels were established for examination, as shown in Table 1. Dealing with Cut Ophiopogonis Radix of different specifications based on factor level is given in Table 1 and L9 [34] orthogonal design table. Table 2 presents the processing techniques employed for Cut Ophiopogonis Radix.

**Table 1:** Factor level table

Levels	Factor (A)	Factor (B)	Factor (C)
1	Cross section	2 mm ≥ thickness > 1 mm	Sun-dried
2	Oblique section	4 mm ≥ thickness > 2 mm	Drying at 55℃ [31]
3	Longitudinal section	6 mm ≥ thickness > 4 mm	Freeze-drying

**Table 2:** Experimental design of Cut Ophiopogonis Radix processing technology

Sample serial number	Experimental design of Cut Ophiopogonis Radix processing technology			
	Processing type	Cutting method	Slicing thickness	Drying method
A_1_B_1_C_1_	Fresh processing	Cross section	2 mm ≥ thickness > 1 mm	Sun-dried
A_1_B_2_C_2_			4 mm ≥ thickness > 2 mm	Drying at 55℃ [31]
A_1_B_3_C_3_			6 mm ≥ thickness > 4 mm	Freeze-drying
A_2_B_1_C_2_		Longitudinal section	2 mm ≥ thickness > 1 mm	Sun-dried
A_2_B_2_C_3_			4 mm ≥ thickness > 2 mm	Drying at 55℃ [31]
A_2_B_3_C_1_			6 mm ≥ thickness > 4 mm	Freeze-drying
A_3_B_1_C_3_		Oblique section	2 mm ≥ thickness > 1 mm	Sun-dried
A_3_B_2_C_1_			4 mm ≥ thickness > 2 mm	Drying at 55℃ [31]
A_3_B_3_C_2_			6 mm ≥ thickness > 4 mm	Freeze-drying

### 3.2 Appearance traits

Cut Ophiopogonis Radix was thoroughly evaluated for its appearance and characteristics with reference to the 2020 edition of the “Pharmacopoeia of the People’s Republic of China” [1]; the appearance shape, surface characteristics, section characteristics, smell, and taste were used as the inspection indicators.

### 3.3 Quality evaluation

#### 3.3.1 Determination method of moisture, total ash, and extract

Samples of Ophiopogonis Radix were analyzed with the 2020 edition of the “Pharmacopoeia of the People’s Republic of China” [1] as a reference for the determination of moisture (General Rule 0832 Second Law), total ash (General Rule 2302), and extract (Cold soaking method under General Chapter 2201).

#### 3.3.2 Determination of total saponins, total flavonoids, and total polysaccharides

Total saponin content was calculated using the procedure described in the section titled “Content Determination of Ophiopogonis Radix” of Volume I of the 2020 edition of the “Pharmacopoeia of the People’s Republic of China” [1]. Total flavonoid content was calculated using the reference technique using hesperidin as the standard [32]. The determination of total polysaccharide content was performed using the reference method, with fructose serving as the reference substance [33,34].

### 3.4 Processing cost

Drying time and energy consumption were used to assess processing costs. The amount of electricity needed to process one batch of one ton of fresh Ophiopogonis Radix was used to determine the amount of energy consumption. Power consumption was calculated as follows: power consumption = electrical power × power consumption time [35].

## 4 Results

### 4.1 Appearance traits of Cut Ophiopogonis Radix

**Figure 1:**
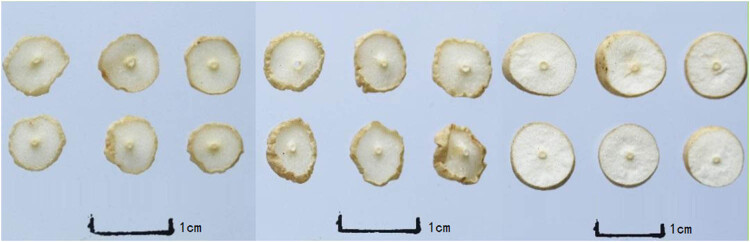
Cut Ophiopogonis Radix (left → right：A1B1C1, A_1_B_2_C_2_, A_1_B_3_C_3_).

**Figure 2:**
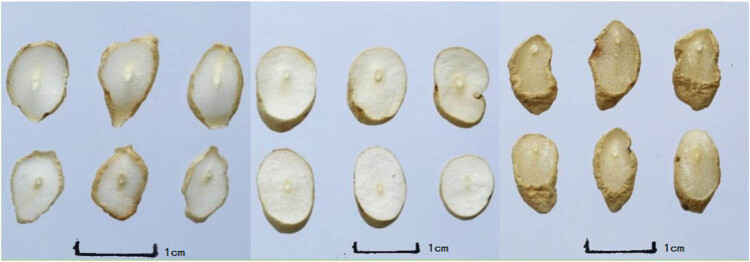
Cut Ophiopogonis Radix (left → right：A_2_B_1_C_2_, A_2_B_2_C_3_, A_2_B_3_C_1_).

**Figure 3:**
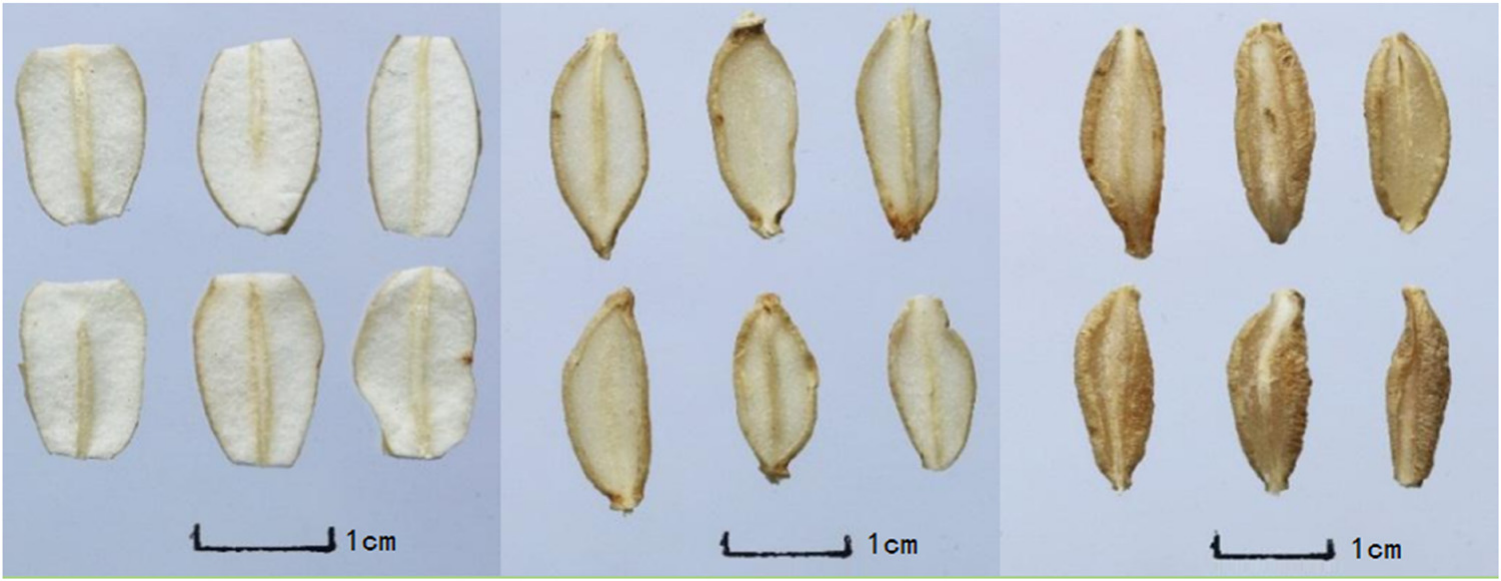
Cut Ophiopogonis Radix (left → right：A_3_B_1_C_3_, A_3_B_2_C_1_, A_3_B_3_C_2_).

There were some changes in the Cut Ophiopogonis Radix generated using various processing techniques in terms of appearance, shape, surface features, section texture, and section color. Slices of Cut Ophiopogonis Radix that had been freeze-dried essentially had the same shape as those that had been cut fresh. However, the Cut Ophiopogonis Radix displayed varying degrees of inward curling following both natural drying and drying at 55°C. Similar observations on the influence of drying methods on the shape of plant fruit were reported, with freeze-drying having no effect but sun and drying at 55°C did [36]. Additionally, the cut surface color of the freeze-dried Cut Ophiopogonis Radix was whiter and lighter than the colors of the sun-dried and 55°C-dried Cut Ophiopogonis Radix, which were both almost white. To summarize, the processes A_1_B_1_C_1_, A_1_B_2_C_2_, and A_3_B_1_C_3_ had superior appearance and properties and can be selected as the best origin of Ophiopogonis Radix as Processing Technology. The shape, color, texture, smell, and taste of various Cut Ophiopogonis Radix after processing are depicted in Table 3 and Figures 1–3.

**Table 3:** Appearance traits of Cut Ophiopogonis Radix

Sample serial number	Appearance shape	Surface features	Cut feature	Smell	Taste
A_1_B_1_C_1_	R, SDC	Epidermis PY, SS	Cut surface Wh, TP	W	S, SB
A_1_B_2_C_2_	R, SDC	Epidermis PY, SS	Cut surface Wh, P	W	S, SB
A_1_B_3_C_3_	R, SDC	Epidermis PY, Sm	Cut surface Wh, LS	W	S, SB
A_2_B_1_C_2_	O, SCC	Epidermis PY, SS	Cut surface Wh, TP	W	S, SB
A_2_B_2_C_3_	O, SCC	Epidermis YW, S	Cut surface Wh, with small white particles	W	S, SB
A_2_B_3_C_1_	Oval, PSC	Epidermis YB, SS	Cut surface Wh, TP	W	S, SB
A_3_B_1_C_3_	S, E, OC	Epidermis YW, SIR	Cut surface Wh, LF, and F	W	S, SB
A_3_B_2_C_1_	S, E, OSC	Epidermis LYB, SI	Cut surface Wh, TP	W	S, SB
A_3_B_3_C_2_	S, E, OSC	Epidermis LYB, with fine Wr, SI	Cut surface Wh, P	W	S, SB

R, round; SDC, small and distinct central column; O, Oval; SCC, small and conspicuous central column; PSC, prominent and small central column; S, spindle; E, elongated; OC, obvious central column; OSC, obvious and small central column; PY, pale yellow; SS, slightly shrunken; Sm, smooth; YW, yellowish white; YB, yellowish-brown; LYB, light yellowish brown; SI, slightly involute; SIR, slightly inwardly rolled; Wr, wrinkles; Wh, white; TP, translucent and powdery; P, powdery; LS, lightly soaked; LF, lightly foamed; F, foamy; W, weak; S, sweet; SB, slightly bitter.

### 4.2 Quality evaluation

#### 4.2.1 Determination of moisture and total ash

The moisture content of Ophiopogonis Radix was assessed through various drying methods. Freeze-drying for 24 h, following General Rule 0832 Second Law, resulted in a moisture content of 42.68 ± 0.67%. Sun-drying yielded a moisture content of 27.34 ± 0.57%, while the drying method at 55°C resulted in a lower moisture content of 22.34 ± 0.63%. The findings indicate that there was no significant disparity (*P*  >  0.05) in the levels of moisture content between the techniques of sun-drying and drying at 55°C. However, a statistically significant difference (*P*  <  0.05) was observed in the moisture content between the methods of freeze-drying and sun-drying/drying at 55°C.

### 4.3 Processing cost of Cut Ophiopogonis Radix

Table 4 displays the results of the processing cost calculation for Cut Ophiopogonis Radix. Based on the table, it can be concluded that A_1_B_2_C_2_ and A_2_B_1_C_2_ processing Cut Ophiopogonis Radix required less drying time and energy than other methods, making them the best option for fresh processing of Cut Ophiopogonis Radix. Similar outcomes were achieved in the chosen processing when drying at 55°C was used with a sample thickness ranging from 2 to 4 mm. Drying periods vary with sample thickness and drying temperature. When the different thicknesses were compared to one another, it was discovered that the specimen with the higher thickness, around 7 mm, displayed delayed drying behavior during the drying process because it contained a high level of moisture, as opposed to the lower thickness, about 5 mm [37,38]. Similar patterns were seen at drying temperatures over 55°C [39].

**Table 4:** The processing cost of Cut Ophiopogonis Radix

Sample serial number	Drying time (h)	Power consumption (kW/h)
A_1_B_1_C_1_	100	0
A_1_B_2_C_2_	16	17.60
A_1_B_3_C_3_	48	39.94
A_2_B_1_C_2_	14	15.40
A_2_B_2_C_3_	48	39.94
A_2_B_3_C_1_	90	0
A_3_B_1_C_3_	48	39.94
A_3_B_2_C_1_	100	0
A_3_B_3_C_2_	28	30.80

### 4.4 Analysis of the results of the Cut Ophiopogonis Radix mass assay

#### 4.4.1 Analysis of Cut Ophiopogonis Radix extract assay results

Table 5 displays the orthogonal test results and visual analysis of Cut Ophiopogonis Radix, while Table 6 displays the variance analysis. The intuitive analysis determined that the order of influence of each factor on the extract was as follows: method of cutting > cutting thickness > drying method. According to the results of the variance analysis, the extract was not significantly affected by the cutting method or cutting thickness (*P* > 0.05); however, it was significantly affected by the drying process (*P* < 0.05). Freeze-drying yielded considerably more extracts from Ophiopogonis Radix medicinal materials after processing by the Cut System compared to other drying processes. As a result, using the extract as the evaluation index, the process A_2_B_2_C_3_ may be utilized as the optimum fresh processing strategy at the source of Cut Ophiopogonis Radix.

**Table 5:** Determination results of extract and visual analysis

Factors	Cutting method	Cutting thickness	Drying method	Extract (%)
A_1_B_1_C_1_	1	1	1	77.15
A_1_B_2_C_2_	1	2	2	78.66
A_1_B_3_C_3_	1	3	3	75.54
A_2_B_1_C_2_	2	1	2	76.64
A_2_B_2_C_3_	2	2	3	83.05
A_2_B_3_C_1_	2	3	1	81.35
A_3_B_1_C_3_	3	1	3	76.72
A_3_B_2_C_1_	3	2	1	76.19
A_3_B_3_C_2_	3	3	2	77.94
Mean 1	77.117	76.837	78.230	
Mean 2	80.347	79.300	77.747	
Mean 3	76.950	78.277	78.437	
Range	3.397	2.463	0.690	

**Table 6:** Analysis of variance of extract results

Source of variance	Sum of square	Degrees of freedom	*F* value	*P*
Cutting method	100.985	8	3.212	0.051
Cutting thickness	0.267	8	0.068	0.800
Drying method	100.718	8	3.661	0.037

#### 4.4.2 Determination results of total saponins in Cut Ophiopogonis Radix

Table 7 displays the findings of the assessment and visual analysis of Cut Ophiopogonis Radix’s total saponin content, while Table 8 presents the results of the variance analysis. According to the results of an intuitive analysis, the order of the influence of each factor on total saponin content was as follows: cutting method > drying method > cutting thickness. The findings of the analysis of variance revealed that the method of cutting, the cutting thickness, and the drying process did not have a significant effect on the total saponin content of the Cut Ophiopogonis Radix (*P* > 0.05).

**Table 7:** Determination results of total saponins and visual analysis

Factors	Cutting method	Cutting thickness	Drying method	Total saponin content (%)
A_1_B_1_C_1_	1	1	1	0.26
A_1_B_2_C_2_	1	2	2	0.23
A_1_B_3_C_3_	1	3	3	0.18
A_2_B_1_C_2_	2	1	2	0.25
A_2_B_2_C_3_	2	2	3	0.24
A_2_B_3_C_1_	2	3	1	0.26
A_3_B_1_C_3_	3	1	3	0.2
A_3_B_2_C_1_	3	2	1	0.2
A_3_B_3_C_2_	3	3	2	0.23
Mean 1	0.223	0.237	0.240	
Mean 2	0.250	0.223	0.237	
Mean 3	0.210	0.223	0.207	
Range	0.040	0.014	0.033	

**Table 8:** Analysis of variance of total saponins results

Source of variance	Sum of square	Degrees of freedom	*F* Value	*P*
Cutting method	0.012	8	1.824	0.194
Cutting thickness	0.001	8	0.795	0.396
Drying method	0.011	8	1.971	0.169

#### 4.4.3 Determination results of total polysaccharide content in Cut Ophiopogonis Radix

Table 9 shows the findings of the determination and visual examination of the total polysaccharide content of Cut Ophiopogonis Radix, and Table 10 shows the results of the variance analysis. According to the findings of the intuitive analysis, the order of the influence of each factor on the total polysaccharide content was as follows: cutting method > cutting thickness > drying method. The findings of the variance analysis demonstrated that the determination of the total polysaccharide content was significantly impacted by the cutting method, cutting thickness, and drying method (*P* < 0.01). After slitting the Ophiopogonis Radix to 1–2 mm (sun-dried) or 2–4 mm (drying at 55°C), the total polysaccharide content was found to be considerably higher than that of other treatments (*P* < 0.05). Previously, the maximum total polysaccharide content was recorded at 60°C, while increasing the drying temperature from 60 to 70°C lowered the total polysaccharide content. This could be because polysaccharides are heat sensitive, and the greater drying temperature resulted in thermal degradation of the polysaccharides [40]. Another study found that sun-drying increased carbohydrate content and that thinner slices (between 1 and 2 mm) were responsible for the decreased moisture content and improved nutrient quality [41]. Consequently, considering the total polysaccharide content as the evaluation index processes A_1_B_1_C_1_ and A_1_B_2_C_2_ can be utilized as the most optimal plan for fresh processing in the origin of Cut Ophiopogonis Radix.

**Table 9:** Determination results of total polysaccharides and visual analysis

Factors	Cutting method	Cutting thickness	Drying method	Total polysaccharide content (%)
A_1_B_1_C_1_	1	1	1	69.58
A_1_B_2_C_2_	1	2	2	64.18
A_1_B_3_C_3_	1	3	3	58.33
A_2_B_1_C_2_	2	1	2	53.11
A_2_B_2_C_3_	2	2	3	57.58
A_2_B_3_C_1_	2	3	1	52.69
A_3_B_1_C_3_	3	1	3	53.27
A_3_B_2_C_1_	3	2	1	53.22
A_3_B_3_C_2_	3	3	2	55.56
Mean 1	64.030	58.653	58.497	
Mean 2	54.460	58.327	57.617	
Mean 3	54.017	55.527	56.393	
Range	10.013	3.126	2.104	

**Table 10:** Analysis of variance of total polysaccharides results

Source of variance	Sum of square	Degrees of freedom	*F* Value	*P*
Cutting method	892.665	8	18.318	0.000
Cutting thickness	347.582	8	57.060	0.000
Drying method	545.082	8	12.783	0.001

#### 4.4.4 Determination results of total flavonoids in Cut Ophiopogonis Radix

Table 11 displays the findings of the determination and visual analysis of Cut Ophiopogonis Radix’s total flavonoid content, while Table 12 presents the results of the variance analysis. According to the results of the intuitive analysis, the order of the influence of each factor on the total flavonoid content was as follows: drying method > cutting method > slicing thickness. The findings of the variance analysis revealed that the method of cutting, the cutting thickness, and the drying process all had an extremely significant influence on the outcomes of the determination of the total flavonoid (*P* less than 0.01). After cutting the Ophiopogonis Radix, the content of total flavonoids was found to be higher when dried in the sun or at a temperature of 55°C than when dried using any other method. As a result, using the total flavonoid content as the evaluation index, procedures A_1_B_1_C_1_ and A_3_B_3_C_2_ can be utilized as the best optimal plan for fresh processing in the origin of Cut Ophiopogonis Radix.

**Table 11:** Determination results of total flavonoids and visual analysis

Factors	Cutting method	Cutting thickness	Drying method	Total flavonoid content (%)
A_1_B_1_C_1_	1	1	1	1.16
A_1_B_2_C_2_	1	2	2	0.81
A_1_B_3_C_3_	1	3	3	0.66
A_2_B_1_C_2_	2	1	2	0.76
A_2_B_2_C_3_	2	2	3	0.78
A_2_B_3_C_1_	2	3	1	0.81
A_3_B_1_C_3_	3	1	3	0.63
A_3_B_2_C_1_	3	2	1	0.78
A_3_B_3_C_2_	3	3	2	0.87
Mean 1	0.877	0.850	0.917	
Mean 2	0.783	0.790	0.813	
Mean 3	0.760	0.780	0.690	
Range	0.117	0.070	0.227	

**Table 12:** Analysis of variance of total flavonoids results

Source of variance	Sum of square	Degrees of freedom	*F* Value	*P*
Cutting method	0.313	8	220.219	0.000
Cutting thickness	0.047	8	263.292	0.000
Drying method	0.266	8	214.065	0.000

## 5 Discussion and conclusion

Ophiopogonis Radix is currently made primarily of steroidal saponins, isoflavones, and polysaccharides. There are numerous biological activities associated with steroidal saponins, high isoflavones, and polysaccharides. Steroidal saponins are the main active site of Ophiopogonis Radix. Total saponins were also employed as indicators in the 2020 edition of the “Pharmacopoeia of the People’s Republic of China” to evaluate the quality indicators of Ophiopogonis Radix medicinal materials and decoction pieces. It has anti-aging, anti-inflammatory, immune-regulating, and anti-cardiovascular and cerebrovascular disease properties as well as improves liver and lung pathological damage [42,43]. Isoflavones at high concentrations have a wide range of biological effects. It is another key component of Ophiopogonis Radix as a specific class of flavonoids with pharmacological activities such as anti-non-small cell lung cancer [44], scavenging oxygen free radicals [45], and cardioprotection [46]. Moreover, the roots of Ophiopogonis Radix are abundant in polysaccharides. The polysaccharides of Ophiopogonis Radix consist of monosaccharides and oligosaccharides, including fructose and numerous oligosaccharides. It has pharmacological effects such as anti-tumor, anti-myocardial ischemia, enhancing immunity, and anti-oxidation [47,48]. The investigation is therefore based on the extracts and total saponins of the quality control indicators for Ophiopogonis Radix in the 2020 edition of the “Pharmacopoeia of the People’s Republic of China.” Total flavonoids and total polysaccharides were incorporated as evaluation indicators to determine the optimal processing technique for Cut Ophiopogonis Radix.

Drying is the most common approach for extending the shelf life of Ophiopogonis Radix by lowering the moisture content to a specific level. It can also reduce material weight and large volumes, lowering transportation and storage costs [49]. The drying temperature has a considerable impact on the drying efficiency and quality of the dried product [50]. Water removal from food products is affected by not only drying temperature [51] but also slice thickness [52]. It is well recognized that the drying conditions, as well as the sample shape, influence the quality of the dried product. After considering the effects of the slicing method, slice thickness, and drying method on the quality of the Cut Ophiopogonis Radix and using the contents of extract, total saponins, total flavonoids, and total polysaccharides as evaluation indexes, the processing technologies A_1_B_1_C_1_, A_1_B_2_C_2_, A_2_B_2_C_3_, and A_3_B_3_C_2_ were determined to be the most effective processing technologies for the Cut Ophiopogonis Radix. The procedure A_1_B_1_C_1_ uses solar drying for a prolonged period; however, because of the huge cut surface, this method is limited by climatic factors and is easily impacted by weather. It is also easily polluted by prolonged exposure to the air. Rainy weather increases the dried product’s susceptibility to rehydration [53,54]. A_2_B_2_C_3_ used a freeze-drying process in which the polysaccharide components were either not cooked at all or just a very tiny portion of the polysaccharide was coked. This resulted in the white color and a lower content of total polysaccharides and total flavonoids in Ophiopogonis Radix than with other drying techniques. The A_3_B_3_C_2_ procedure was utilized to process the medicinal material Ophiopogonis Radix. The material was subjected to bevel cutting, resulting in slightly inwardly rolled slices that were prone to warping. The procedure aimed to combine the material’s properties, including appearance characteristics, processing cost, and intrinsic quality, after the cutting process. Furthermore, as per the 2020 edition of the “Pharmacopoeia of The People’s Republic of China,” the most favored technique for processing Cut Ophiopogonis Radix was found to be the A_1_B_2_C_2_ process (which involves horizontally slicing Fresh Ophiopogonis Radix to a thickness of 4 mm or more but less than 2 mm, followed by drying at a temperature of 55℃ or lower). As previously reported, an investigation was conducted on the relationship between the moisture content of a sample and its drying time, with varying sample thicknesses at a drying temperature of 55°C. The results indicated that lower thicknesses and the aforementioned temperature yielded more favorable outcomes. Conversely, greater sample thicknesses necessitated a lengthier drying time due to the increased distance that moisture had to travel to reach the surface. Comparable patterns were likewise noted at drying temperatures of 60 and 65°C [32]. The processing procedure exhibited a low cost of processing and high efficiency of drying, resulting in the production of Cut Ophiopogonis Radix which lacked any noticeable warped flakes, possessed a smooth flake shape, and contained a high concentration of active ingredients. These findings provide additional evidence supporting the viability of fresh processing Cut Ophiopogonis Radix.

In the context of the conventional production process of decoction pieces, the majority of traditional Chinese medicines undergo processing into dry medicinal materials at their place of origin, followed by transportation to various locations where they are subjected to processes such as infiltration, cutting, and drying, ultimately resulting in the formation of Chinese medicine decoction pieces. The repeated infiltration, drying, and other secondary processing result in the destruction and loss of active ingredients, as well as an increase in production costs. The process of fresh processing in the production of Chinese medicinal materials plays a crucial role in maintaining their quality, particularly in the case of Chinese herbal decoction pieces. This process serves as the first step in ensuring the overall quality of medicines. The integration of fresh processing and processing in the origin of Chinese herbal medicines can combine the production specifications of Chinese herbal medicines with the production specifications of decoction pieces, thereby facilitating the maintenance of the quality and clinical effectiveness of Chinese herbal medicine pieces.
